# Multicenter Analysis of Liver Injury Patterns and Mortality in COVID-19

**DOI:** 10.3389/fmed.2020.584342

**Published:** 2020-10-20

**Authors:** Huikuan Chu, Tao Bai, Liuying Chen, Lilin Hu, Li Xiao, Lin Yao, Rui Zhu, Xiaohui Niu, Zhonglin Li, Lei Zhang, Chaoqun Han, Shuangning Song, Qi He, Ying Zhao, Qingjing Zhu, Hua Chen, Bernd Schnabl, Ling Yang, Xiaohua Hou

**Affiliations:** ^1^Division of Gastroenterology, Union Hospital, Tongji Medical College, Huazhong University of Science and Technology, Wuhan, China; ^2^Department of Integrated Chinese and Western Medicine, Union Hospital, Tongji Medical College, Huazhong University of Science and Technology, Wuhan, China; ^3^College of Informatics, Huazhong Agricultural University, Wuhan, China; ^4^Liver and Infectious Diseases Department, Wuhan Jinyintan Hospital, Wuhan, China; ^5^Tuberculosis and Respiratory Department, Wuhan Jinyintan Hospital, Wuhan, China; ^6^Department of Medicine, University of California, San Diego, La Jolla, CA, United States

**Keywords:** liver impairment, hepatocellular pattern, cholestatic pattern, mixed pattern, prognosis

## Abstract

**Background and Aim:** Liver test abnormalities are common in COVID-19 patients. The aim of our study was to determine risk factors for different liver injury patterns and to evaluate the relationship between liver injury patterns and prognosis in patients with COVID-19.

**Methods:** We retrospectively analyzed patients admitted between January 1st to March 10th, with laboratory-confirmed COVID-19 and followed them up to April 20th, 2020. Information of clinical features of patients was collected for analysis.

**Results:** As a result, a total of 838 hospitalized patients with confirmed COVID-19, including 48.8% (409/838) patients with normal liver function and 51.2% (429/838) patients with liver injury were analyzed. Abnormal liver function tests are associated with organ injuries, hypoxia, inflammation, and the use of antiviral drugs. Hepatocellular injury pattern was associated with hypoxia. The mortality of the hepatocellular injury pattern, cholestatic pattern and mixed pattern were 25, 28.2, and 22.3%, respectively, while the death rate was only 6.1% in the patients without liver injury. Multivariate analyses showed that liver injury with cholestatic pattern and mixed pattern were associated with increased mortality risk.

**Conclusions:** Our study confirmed that hepatocellular injury pattern that may be induced by hypoxia was not risk factor for mortality in SARS-COV-2 infection, while liver injury with mixed pattern and cholestatic pattern that might be induced by SARS-CoV-2 directly might be potential risk factors for increased mortality in COVID-19 patients.

## Introduction

The severe acute respiratory syndrome coronavirus 2 (SARS-Cov-2) disease, which is also called Corona Virus Disease 2019 (COVID-19), has been considered as a public health emergency of international concern by World Health Organization (WHO). Currently, SARS-CoV-2 has spread to over 200 countries and areas with 11, 327, 790 confirmed cases, including 532, 340 deaths globally until July 6 (1). Although COVID-19 mainly affects the lower respiratory tract and manifests as pneumonia in humans, a subset of COVID-19 patients present with different degrees of liver injury (2–4).

On the basis of previous reports from China, 15–26% of COVID-19 patients develop severe pneumonia with increased mortality (5). Organ dysfunction including acute kidney injury and liver injury is common in patients with severe pneumonia (5–8). 16–53% of COVID-19 patients had liver injury with abnormal levels of alanine aminotransferase (ALT) and/or aspartate aminotransferase (AST) accompanied by slightly elevated bilirubin levels during disease progression (2–4). The incidence of liver injury in COVID-19 patients with severe disease (62%) was significantly higher than that in patients with mild disease (25%) (4, 9). In addition, hepatic steatosis and liver injury were also confirmed by liver biopsy from a deceased patient infected with COVID-19 (10), and 78% of deceased COVID-19 patients had liver injury (11). However, whether liver injury is related with the progression of COVID-19 remains controversial (12–14). Besides, liver injury with hepatocyte pattern or mixed pattern had significantly higher risks of developing severe pneumonia in a single-center study of 148 patients (13), while the risk factors for different liver injury patterns remain unclear and a large-scale, multicenter study on the detailed relationships between different liver injury patterns and progression of COVID-19 was absent. In this study, we analyzed data from 838 hospitalized COVID-19 patients in multiple centers, explored the risk factors of liver injury among COVID-19 patients and evaluated the relationship between different patterns of liver injury and the progression of COVID-19. We found that liver injury was closely related with organ injuries, hypoxia, inflammation and the utilization of antiviral drugs, hepatocellular injury pattern that was associated with hypoxia was not risk factor for increased mortality, while liver injury with cholestatic pattern and mixed pattern that may be induced by SARS-CoV-2 directly increased mortality risk for COVID-19 patients.

## Methods

### Study Design and Participants

This study was conducted in accordance with the principles of the Declaration of Helsinki and approved by the Institutional Ethics Board of Union Hospital, Tongji Medical College, Huazhong University of Science and Technology, and Jinyintan Hospital in China. We obtained the clinical and laboratory data at admission of confirmed COVID-19 patients from two centers including Union Hospital, Tongji Medical College, Huazhong University of Science and Technology, and Jinyintan Hospital that were designated hospitals in Wuhan to manage patients with COVID-19. We retrospectively analyzed patients admitted between January 1st to March 10th, 2020, and followed them up to April 20th, 2020, with laboratory-confirmed COVID-19 based on real-time reverse-transcriptase polymerase-chain-reaction (RT-PCR) assay for nasal and pharyngeal swab specimens (9). The inclusion criteria were: 1. Patients confirmed with COVID-19; 2. Adult patients aged >18 years; 3. Patients had definite clinical outcome (cured or died) before April 20th. Exclusion criteria were: 1. Patients had pre-existing liver disease; 2. Patients aged <18 years. All patients with COVID-19 enrolled in this study were diagnosed according to The World Health Organization Interim Guidance.

### Data Collection

The medical records of patients were collected and analyzed by the search team from Union Hospital, Tongji Medical College, Huazhong University of Science and Technology, and Jinyintan Hospital. The clinical symptoms on admission, vital signs, and the pre-existing comorbidities of the patients were obtained from the electronic medical records. The results of the laboratory and imaging examinations on admission were obtained from the electronic system of laboratory and imaging examination. Diagnoses of acute kidney injuries and cardiac injuries were defined according to the Berlin Definition, improving global outcome definition and elevated cardiac biomarker, respectively (10, 15, 16). The degree of severity of COVID-19 (severe vs. non-severe) was defined using the American Thoracic Society guidelines for community-acquired pneumonia (17). For the diagnosis of three patterns of liver injury, initially ALT activity [patients ALT/upper limit of normal (ULN) of ALT] and alkaline phosphatase (ALP) activity (patients ALP/ULN of ALP) is calculated. Then ALT/ALP ratio (R) is determined. Hepatocellular pattern: If ALT alone is elevated ≥5-fold above ULN or R ≥5. Cholestatic pattern: ALP alone is elevated ≥2-fold above ULN or R ≤ 2. Mixed pattern: R >2 to <5 (18, 19).

### Statistical Analysis

Categorical variables were presented as numbers and percentages. Chi-square tests with Bonferoni corrections for intragroup comparisons and Fisher's exact tests were used for categorical variables. Continuous values were expressed as means (standard deviations), and were calculated using the Student's *t-*test or one-way ANOVA for parametric data. Continuous values were expressed as median [interquartile range (IQR)], and were calculated using the Mann–Whitney U or Kruskal-Wallis H test with Bonferoni corrections for intragroup comparisons for non-parametric data. The correlation of hypoxia/inflammation and abnormal liver function test was examined using Spearman Rho's (ρ) correlation. To identify the risk factors for poor outcomes, univariate and multivariate Cox regression analyses were performed and reported as hazard ratios (HRs) and 95% confidence intervals (95% CIs). All statistical analyses were performed using SPSS (Statistical Package for the Social Sciences) (version 13.0, IBM Corp, Armonk, NY, USA). A significance level of *P* ≤ 0.05 was used for all models (two-sided).

## Results

### Clinical Characteristics of Patients

**Figure 1:**
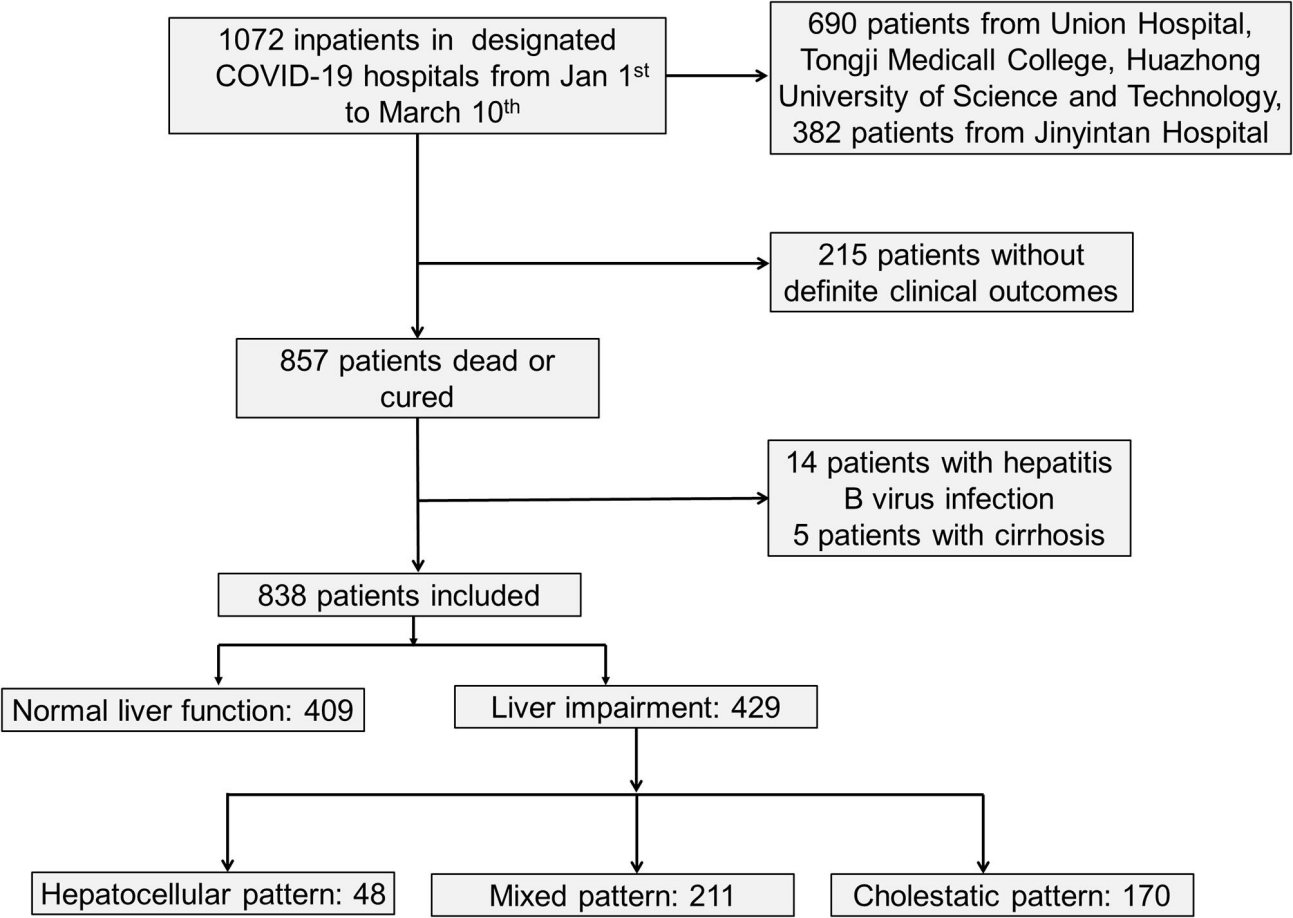
Selection diagram of included patients.

As shown in Figure 1 and Table 1, a total of 838 hospitalized patients with confirmed COVID-19, including 48.8% (409/838) patients with normal liver function and 51.2% (429/838) patients with liver injury were analyzed. A total of 65.3% (280/429) male patients had liver injury, while in female patients, this proportion was significantly lower. The average age for patients with liver injury was 61, which is older than that in patients with normal liver function. In addition, more patients with liver injury had severe COVID-19. These results indicated that male and older patients and patients with more severe disease were more susceptible to liver injury.

**Table 1:** Clinical and laboratory characteristics of patients with COVID-19.

**Variables**	**Normal liver function**	**Liver injury**	***P*-values**
	**n (%) or median (IQR)**	**n (%) or median (IQR)**	
Sex			^***^
Male	184 (45.0)	280 (65.3)	
Female	225 (55.0)	149 (34.7)	
Age (years)	56 (43–66)	61 (49–69)	^***^
Severe	45 (11.0)	141 (32.9)	^***^
Hypertension	105 (25.7)	162 (37.8)	^***^
Diabetes	57 (13.9)	71 (16.6)	0.293
Coronary heart disease	24 (5.9)	41 (9.6)	^*^
Chronic kidney disease	7 (1.7)	12 (2.8)	0.291
Malignant tumor	8 (2.0)	20 (4.7)	^*^
Blood oxygen saturation (%)	97 (95–98)	95 (91–98)	^***^
White blood cell (10^9^/L)	5.4 (4.1–6.8)	6.5 (4.8–9.0)	^***^
Lymphocyte (10^9^/L)	1.2 (0.8–1.6)	0.9 (0.6–1.4)	^***^
Neutrophils (10^9^/L)	3.6 (2.6–4.9)	5.1 (3.4–7.6)	^***^
Hemoglobin	125 (114–136)	130 (119–140)	^***^
Platelets (10^9^/L)	204 (160–268)	205 (148–280)	0.806
Liver function test			
Total bilirubin (μmol/L)	9.6 (7.0–11.9)	13.2 (9.6–20.0)	^***^
Alanine aminotransferase (U/L)	19 (14–26)	49 (32–71)	^***^
Aspartate aminotransferase (U/L)	23 (18–29)	44 (31–59)	^***^
Alkaline phosphatase (U/L)	58 (47–70)	69 (52–95)	^***^
Gamma glutamyltransferase (U/L)	22 (15–31)	49 (26–93)	^***^
Albumin (g/L)	34.4 (30.0–38.5)	30.8 (27.1–35.2)	^***^
Creatinine (μmol/L)	65.0 (54.0–77.0)	72.7 (60.7–88.5)	^***^
Prothrombin time (s)	12.9 (11.8–13.7)	13.1 (12.0–14.3)	^**^
Activated partial thromboplastin time (s)	36.6 (34.0–38.8)	36.5 (32.6–40.2)	0.780
D-dimer (mg/L)	0.43 (0.23–0.98)	0.80 (0.41–3.06)	^***^
International normalized ratio	0.98 (0.93–1.06)	1.00 (0.94–1.09)	^**^
C-reactive protein (mg/L)	10.6 (2.9–41.8)	41.7 (9.1–90.5)	^***^
Procalcitonin (ug/L)	0.05 (0.04–0.09)	0.09 (0.05–0.23)	^***^
Ferritin (ug/L)	281.3 (129.0–567.4)	651.2 (329.0–1390.4)	^***^
Troponin I (μg/L)	3.2 (1.4–7.5)	5.4 (2.3–20.1)	^***^
Creatine Kinase (U/L)	67 (46–115)	99 (53–228)	^***^
Cardiac injury	18 (4.4)	77 (17.9)	^***^
Kidney injury	8 (2.0)	20 (4.7)	^*^
Systemic inflammatory response syndrome	79 (19.3)	114 (26.6)	^*^

* *P < 0.05*,

** *P < 0.01*,

*** *P < 0.001. ULN, upper limit of normal; IQR, interquartile range*.

Among them, 31.8% (267/838) had hypertension, 15.3% (128/838) had diabetes, and 7.8% (65/838) had coronary heart diseases (Table 1). A higher proportion of COVID-19 patients with liver injury had pre-existing hypertension, coronary heart diseases and malignant tumor, while there was no difference in pre-existing diabetes or chronic renal diseases between the subgroups with normal liver function and liver injury (Table 1).

### Liver Biochemical Abnormalities Were Related With Organ Injuries

We evaluated the relationship between liver biochemical abnormalities and other laboratory results in COVID-19 patients. Patients with liver biochemical abnormalities showed more severe lymphocytopenia with lower counts of lymphocytes, more inflammation as indicated by elevated level of serum white blood cells, C-reactive protein (CRP) and procalcitonin (PCT), higher level of D-dimers, kidney injury as indicated by elevated level of serum creatinine, and higher level of serum ferritin. Importantly, patients with liver biochemical abnormalities had a higher incidence of cardiac injury, kidney injury, and systemic inflammatory response syndrome (SIRS) (Table 1). These results indicated that liver biochemical abnormalities were closely associated with cardiac injury, kidney injury and systemic inflammatory response that play important roles in COVID-19 patients.

### Liver Biochemical Abnormalities Were Closely Associated With Hypoxia/Inflammation

Hypoxia and inflammatory cytokine storm are considered as risk factor for liver biochemical abnormalities in patients with COVID-19 (20). Therefore, we analyzed the relationship between liver biochemical abnormalities and hypoxia or inflammatory cytokines. As shown in Table 1, patients with liver injury had much lower blood oxygen saturation. Correlation analysis showed that blood oxygen saturation was negatively related with ALT, AST, TBIL, ALP, and GGT indicating that hypoxia might contribute to liver injury (Table 2).

**Table 2:** Correlation between hypoxia/inflammation and liver biochemical abnormalities in COVID-19.

		**ALT**	**AST**	**TBIL**	**ALP**	**GGT**
Blood oxygen saturation (%)	rho	−0.229	−0.375	−0.173	−0.136	−0.226
	*P*-value	^***^	^***^	^***^	^***^	^***^
IL-2	rho	−0.299	−0.237	0.134	−0.328	−0.109
	*P*-value	0.177	0.288	0.551	0.136	0.628
IL-4	rho	−0.278	−0.200	0.194	−0.071	−0.026
	*P*-value	0.211	0.373	0.387	0.755	0.906
IL-6	rho	0.115	0.223	0.289	0.182	0.094
	*P*-value	0.052	^***^	^***^	^**^	0.114
IL-8	rho	0.136	0.363	0.103	0.129	0.104
	*P*-value	0.277	^**^	0.412	0.304	0.405
IL-10	rho	−0.017	0.146	−0.131	−0.053	0.055
	*P*-value	0.876	0.176	0.222	0.622	0.609
TNF-alpha	rho	0.216	0.307	−0.006	0.160	0.246
	*P*-value	0.043	^**^	0.958	0.136	^*^

*Data are given as Spearman's rho correlation coefficients and P-value*.

* *P < 0.05*,

** *P < 0.01*,

*** *P < 0.001*.

*ALP, alkaline phosphatase; ALT, alanine aminotransferase; AST, aspartate aminotransferase; GGT, gamma glutamyltransferase; IL-2, Interleukin 2; TBIL, Total bilirubin*.

To determine which inflammatory cytokine is associated with liver injury, we measured serum level of Interleukin (IL)-2, IL-4, IL-6, IL-8, IL-10, and tumor necrosis factor-α (TNF-α) in patients with normal liver function and with liver injury. As shown in Supplementary Table 1, serum IL-6 and TNF-α were significantly increased in the liver injury group. IL-6 was positively correlated with the increased AST, TBIL, and ALP. IL-8 was positively correlated with increased AST, and TNF-α was positively correlated with increased ALT, AST, and GGT (Table 2). These results indicate that inflammatory cytokines might contribute to liver injury in patients with COVID-19.

### Lopinavir/Litonavir and Ribavirin Increased the Risk of Liver Biochemical Abnormalities

Antiviral drugs recommended to treat COVID-19 include umifenovir (arbidol hydrochloride), lopinavir/litonavir, ribavirin and interferon, which are metabolized in liver and can induce hepatotoxicity. We tested an association of antiviral drug use and liver injury in COVID-19 patients. As shown in Table 3, the level of ALT, AST, ALP, GGT, and TBil showed no significant difference between patients with and without umifenovir treatment. Patients treated with lopinavir/litonavir had higher levels of AST and GGT (Table 3). In addition, use of ribavirin slightly increased the level of ALP (Table 3). These results indicate that there is an association between the use of lopinavir /litonavir and ribavirin as the antiviral drugs and increased liver injury in COVID-19 patients.

**Table 3:** The association of anti-viral treatment and abnormal liver function test in COVID-19.

	**ALT (U/L)**	**AST (U/L)**	**TBIL (μmol/L)**	**ALP (U/L)**	**GGT (U/L)**	
Umifenovir	No	34 (20–59)	26 (18–75)	10.6 (8.6–13.6)	54 (47–89)	34 (19–103)
	Yes	34 (20–54)	31 (23–46)	10.7 (8.0–14.3)	53 (43–71)	29 (19–54)
	*P*-value	0.670	0.826	0.857	0.174	0.417
Lopinavir /litonavir	No	33 (20–52)	29 (22–45)	10.7 (8.1–14.0)	53 (43–69)	28 (19–52)
	Yes	41 (23–68)	39 (29–69)	9.8 (7.6–15.2)	52 (42–73)	45 (24–79)
	*P*-value	0.060	^**^	0.744	0.893	^**^
Ribavirin	No	34 (21–56)	32 (23–47)	10.6 (8.0–14.0)	52 (42–69)	29 (19–54)
	Yes	34 (20–64)	26 (20–46)	11.6 (8.0–15.3)	56 (49–87)	41 (22–78)
	*P*-value	0.994	0.288	0.526	^*^	0.186
Interferon	No	34 (21–57)	32 (23–47)	10.8 (8.1–14.6)	54 (43–71)	30 (20–56)
	Yes	32 (19–46)	30 (23–44)	10.1 (7.7–12.8)	49 (41–61)	27 (19–53)
	*P*-value	0.120	0.577	0.251	^*^	0.283

*Data for the level of ALT, AST, TBIL, ALP, and GGT were expressed as median (interquartile range)*.

* *P < 0.05*,

** *P < 0.01. ALP, alkaline phosphatase; ALT, alanine aminotransferase; AST, aspartate aminotransferase; GGT, gamma glutamyltransferase; TBIL, Total bilirubin*.

### Hepatocellular Injury Pattern Was Closely Related With Hypoxia

To further understand the characteristic of liver injury in patients with COVID-19, we classified the liver biochemical abnormalities as hepatocellular pattern, cholestatic pattern and mixed pattern according to the ALT/ALP ratio. Most patients (49.2%) manifested with a mixed liver injury pattern (211/429), and 39.6% of patients presented with cholestatic pattern (170/429). Hepatocellular pattern only accounted for 11.2% of patients (48/429). There was no difference between these three liver injury patterns in terms of gender, age, severity of COVID-19 and pre-existing diseases (Supplementary Table 2).

We further evaluated the relationship between different liver injury patterns and laboratory parameters (Supplementary Table 2). Patients with a hepatocellular injury pattern had lower blood oxygen saturation, higher ferritin level and increased kidney injury (Supplementary Table 2). These results indicated that hypoxia might play a critical role in hepatocytes death in COVID-19 patients.

### The Association Between Liver Injury and COVID-19 Severity

We finally evaluated the relationship between different patterns of liver injury and COVID-19 outcome (Table 4). It was worth noting that 47.9, 29.4, and 32.2% of COVID-19 patients with hepatocellular pattern, cholestatic pattern and mixed pattern, respectively, were severe cases compared with 11% of patients with normal liver function (Table 4). The death rates of the COVID-19 patients with hepatocellular pattern, cholestatic pattern, and mixed pattern were 25, 28.2, and 22.3%, respectively, compared with 6.1% of patients with normal liver function (Table 4).

**Table 4:** Relationship between liver injury pattern and disease progress.

**Variables**	**No liver injury (*n =* 409)**	**Hepatocellular pattern (*n =* 48)**	**Cholestatic pattern (*n =* 170)**	**Mixed pattern (n = 211)**	***P_**1**_***	***P_**2**_***	***P_**3**_***
	**n (%) or median (IQR)**	**n (%) or median (IQR)**	**n (%) or median (IQR)**	**n (%) or median (IQR)**			
D-dimer (mg/L)	0.43 (0.23–0.98)	0.83 (0.42–2.62)	0.85 (0.43–4.94)	0.72 (0.41–2.33)	^***^	^***^	^***^
International normalized ratio	0.98 (0.93–1.06)	0.99 (0.94–1.06)	1.01 (0.94–1.13)	1.00 (0.93–1.09)	0.495	^**^	0.088
C-reactive protein (mg/L)	10.6 (2.9–41.8)	40.8 (14.2–102.1)	46.4 (8.9–104.9)	39.2 (8.0–79.6)	^***^	^***^	^***^
Procalcitonin (ug/L)	0.05 (0.04–0.09)	0.12 (0.05–0.41)	0.09 (0.05–0.23)	0.09 (0.05–0.23)	^***^	^***^	^***^
Ferritin (ug/L)	281.3 (129.0–567.4)	1171.8 (601.8–2000.0)	636.5 (351.9–1543.7)	579.8 (284.1–1257.8)	^***^	^***^	^***^
Cardiac injury	18 (4.4)	12 (25.0)	32 (18.8)	33 (15.6)	^***^	^***^	^***^
Kidney injury	8 (2.0)	5 (10.4)	10 (5.9)	5 (2.4)	^**^	^*^	0.771
Systemic inflammatory response syndrome	79 (19.3)	19 (39.6)	47 (27.6)	48 (22.7)	^***^	^*^	0.316
Severe	45 (11.0)	23 (47.9)	50 (29.4)	68 (32.2)	^***^	^***^	^***^
Deceased	25 (6.1)	12 (25.0)	48 (28.2)	47 (22.3)	^***^	^***^	^***^

*P_1_, Hepatocellular pattern vs. no liver injury; P_2_, Cholestatic pattern vs. no liver injury; P_3_, Mixed pattern vs. no liver injury*.

* *P < 0.05*,

** *P < 0.01*,

*** *P < 0.001. IQR, interquartile range*.

Multivariate analyses showed that the liver injury was associated with increased mortality risk in patients with COVID-19, with an adjusted hazard ratio of 2.65 (1.22–5.76) compared with normal liver function (Table 5). In addition, other factors such as platelet count and coagulation factors that represent the state of liver's ability to function were also associated with increased mortality risk in patients with COVID-19 (Supplementary Tables 3, 4). The levels of platelet count decreased in deceased patients with COVID-19, while prothrombin time, D-dimer and international normalized ratio increased in deceased patients with COVID-19 (Supplementary Table 3). Further analysis showed that liver injury with cholestatic pattern and mixed pattern were associated with increased mortality risks in patients with COVID-19 (Table 5). These results indicate that liver injury with cholestatic pattern and mixed pattern are associated with worse prognosis in patients with COVID-19.

**Table 5:** Multivariate Cox regression analyses of liver injury association with mortality.

**Variables**	**HR**	**95%CI**	***P-*value**
liver injury	2.65	1.22–5.76	^*^
Hepatocellular pattern	1.74	0.61–4.97	0.301
Cholestatic pattern	3.05	1.29–7.22	^*^
Mixed pattern	2.70	1.19–6.15	^*^

*P-values were adjusted by age, sex, on admission blood oxygen saturation, kidney injury or cardiac injury*.

* *P < 0.05. CI, confidence interval; HR, Hazard ratio*.

## Discussion

Severe respiratory failure is more likely to be the main cause of mortality in COVID-19 patients (21). Other potential factors such as older age, high Sequential Organ Failure Assessment (SOFA) score, and d-dimer > 1 μg/mL were also associated with poor prognosis (22). On the basis of previous reports from China, some COVID-19 patients present with liver injury (7, 8, 23). Since subtypes of liver injury include hepatocellular pattern, cholestatic pattern, and mixed pattern, we mainly evaluated the incidence of three subtypes in COVID-19 patients and assessed their relationship with prognosis of COVID-19. In this study, we found that liver injury with cholestatic and mixed pattern were associated with worse prognosis of patients with COVID-19.

Our data indicate that SARS-CoV-2 associated liver injury correlates with hypoxia/ inflammation. The mechanism by which COVID-19 patients present with liver injury is still unclear. It was reported that SARS-CoV-2 uses angiotensin converting enzyme II (ACE2) for cell entry (24). Apart from the lung alveolar epithelial cells, liver cholangiocytes also express of ACE2 (24, 25). Besides, coronavirus particles were also identified in the liver of two deceased SARS-CoV-2 patients (26). These suggest that SARS-CoV-2 may damage cholangiocytes directly and lead to cholestatic and mixed pattern liver injury in COVID-19 patients. Interestingly, viral inclusions were not observed in the liver biopsy specimens of some COVID-19 patient with liver injury (10), indicating that SARS-CoV-2 may induce liver injury through other mechanisms. Inflammation may also contribute to liver injury as patients with liver injury had higher level of inflammatory markers, such as CRP and PCT (9, 27). Wang et al. (28) confirmed that the inflammatory storm is closely related to multiple organ damage or death in COVID-19 patients. Our data also confirmed that liver injury correlates with inflammatory cytokines. In addition, hypoxia presenting as one of the common symptoms in COVID-19 patients, may be also related with liver injury (29). Our data show that liver injury is negatively correlated with blood oxygen saturation. Adverse effect of some drugs may be another factor to cause liver injury (30, 31). The use of lopinavir/ritonavir before admission was significantly higher in patients with emerging liver injury than that in patients with normal liver functions, suggesting that application of antiviral drugs may lead to liver injury during the process of COVID-19 treatment (27). Our data confirmed that liver injury for COVID-19 patients was associated with the use of certain antiviral drugs, such as lopinavir /litonavir and ribavirin.

Our data show that patients with liver injury had higher level of serum ferritin and more severe hypoxia especially for patients with hepatocellular injury pattern. It was reported that SARS-CoV-2 destroys hemoglobin in red blood cells, dissociating iron and deoxyhemoglobin, causing less hemoglobin that can carry oxygen, and producing symptoms of respiratory distress and hypoxia (32). Besides, iron dissociated from heme will be stored in ferritin, thus the level of ferritin in serum will be increased (32). In addition, hypoxia may also induce liver injury (29), and damage hepatocytes. Therefore, we propose that the elevated level of serum ferritin and hypoxia are related with hepatocyte injury.

Similar to other reports (6, 33), our study showed that liver injury in COVID-19 patients was frequent but mild in nature. The patterns of liver injury were mostly cholestatic and mixed pattern, which maybe induced by SARS-CoV-2 directly. Besides, liver injury with cholestatic and mixed patterns were associated with the worse prognosis of COVID-19. Thus, clinical physicians should pay more attention to COVID-19 patients with cholestatic and mixed pattern liver injury and give effective treatment method.

Our study has some limitations. Liver injury in COVID-19 is associated with hepatotoxic drug intake and tissue hypoxia. Therefore, increased liver injury induced by hypoxia, or hepatotoxic drug intake may not be reflective of risk factors for mortality in SARS-COV-2 infection. Although large enough to conduct valid comparisons among groups, the sample size remains limited. Larger studies should be performed to further evaluate the predictive value of liver injury for prognosis of COVID-19. In addition, this was a retrospective study, and an unknown bias may exist.

In summary, our study demonstrates that liver injury is closely related with organ injuries, hypoxia, inflammation, and the use of antiviral drugs. Hepatocellular injury pattern is associated with hypoxia, and increased hepatocellular injury may not be risk factor for mortality in SARS-COV-2 infection, while liver injury with cholestatic, and mixed patterns that may be induced by SARS-CoV-2 directly may be potential risk factors for increased mortality in patients with COVID-19. COVID-19 patients with liver injury especially for cholestatic and mixed patterns should be monitored and evaluated frequently and performed effective treatment.

## Data Availability Statement

All datasets generated for this study are included in the article/Supplementary Material.

## Ethics Statement

The studies involving human participants were reviewed and approved by Union Hospital, Tongji Medical College, Huazhong University of Science and Technology, and Jinyintan Hospital. Written informed consent for participation was not required for this study in accordance with the national legislation and the institutional requirements.

## Author Contributions

HChu was responsible for study concept and design, analysis and interpretation of data, and drafting of the manuscript. TB was responsible for acquisition, analysis of data, and critical revision of the manuscript. LH, LX, LYao, RZ, ZL, LZ, CH, SS, QH, and YZ provided assistance in data acquisition and revision of the manuscript. LC and XN provided assistance in data analysis and revision of the manuscript. QZ, HChe, and BS provided critical revision of the manuscript. LYan and XH was responsible for the study concept and design, critical revision of the manuscript, and study supervision. All authors contributed to the article and approved the submitted version.

## Conflict of Interest

The authors declare that the research was conducted in the absence of any commercial or financial relationships that could be construed as a potential conflict of interest.

## Abbreviations

ACE2: angiotensin converting enzyme II
ALP: alkaline phosphatase
ALT: alanine aminotransferase
AST: aspartate aminotransferase
Cis: confidence intervals
COVID-19: Corona Virus Disease 2019
CRP: C-reactive protein
GGT: gamma glutamyltransferase
HRs: hazard ratios
IL-6: Interleukin 6
IQR: interquartile range
ORs: odds ratios
PCT: procalcitonin
R: ratio
RT-PCR: real-time reverse-transcriptase polymerase-chain-reaction
SARS-Cov-2: severe acute respiratory syndrome coronavirus 2
SIRS: systemic inflammatory response syndrome
TNF-α: tumor necrosis factor-α
WHO: World Health Organization
ULN: upper limit of normal.
