# Corrigendum: Multicenter Analysis of Liver Injury Patterns and Mortality in COVID-19

**DOI:** 10.3389/fmed.2021.764493

**Published:** 2021-09-16

**Authors:** Huikuan Chu, Tao Bai, Liuying Chen, Lilin Hu, Li Xiao, Lin Yao, Rui Zhu, Xiaohui Niu, Zhonglin Li, Lei Zhang, Chaoqun Han, Shuangning Song, Qi He, Ying Zhao, Qingjing Zhu, Hua Chen, Bernd Schnabl, Ling Yang, Xiaohua Hou

**Affiliations:** ^1^Division of Gastroenterology, Union Hospital, Tongji Medical College, Huazhong University of Science and Technology, Wuhan, China; ^2^Department of Integrated Chinese and Western Medicine, Union Hospital, Tongji Medical College, Huazhong University of Science and Technology, Wuhan, China; ^3^College of Informatics, Huazhong Agricultural University, Wuhan, China; ^4^Liver and Infectious Diseases Department, Wuhan Jinyintan Hospital, Wuhan, China; ^5^Tuberculosis and Respiratory Department, Wuhan Jinyintan Hospital, Wuhan, China; ^6^Department of Medicine, University of California, San Diego, La Jolla, CA, United States

**Keywords:** liver impairment, hepatocellular pattern, cholestatic pattern, mixed pattern, prognosis

In the published article, there was an error in affiliation ^**^1^**^. Instead of “Division of Gastroenterology, Tongji Medical College, Union Hospital, Huazhong University of Science and Technology, Wuhan, China,” it should be “Division of Gastroenterology, Union Hospital, Tongji Medical College, Huazhong University of Science and Technology, Wuhan, China.”

In the published article, there was an error in affiliation ^**^2^**^. Instead of “Department of Integrated Chinese and Western Medicine, Tongji Medical College, Union Hospital, Huazhong University of Science and Technology, Wuhan, China,” it should be “Department of Integrated Chinese and Western Medicine, Union Hospital, Tongji Medical College, Huazhong University of Science and Technology, Wuhan, China.”

The authors apologize for this error and state that this does not change the scientific conclusions of the article in any way. The original article has been updated.

## Publisher's Note

All claims expressed in this article are solely those of the authors and do not necessarily represent those of their affiliated organizations, or those of the publisher, the editors and the reviewers. Any product that may be evaluated in this article, or claim that may be made by its manufacturer, is not guaranteed or endorsed by the publisher.

