# Supplementary material for: Multicenter Analysis of Liver Injury Patterns and Mortality in COVID-19
Source: Front Med (Lausanne). 2020 Oct 20;7:584342. doi: 10.3389/fmed.2020.584342 (PMC7606271; doi:10.3389/fmed.2020.584342)
Supplement: Supplementary file 1 [file Data_Sheet_1.docx]

**Multicenter analysis of liver injury patterns and mortality in COVID-19**

Huikuan Chu^#1^, Tao Bai^#1^, Liuying Chen^1^, Lilin Hu^1^, Li Xiao^1^, Lin Yao^1^, Rui Zhu^2^, Xiaohui Niu^3^, Zhonglin Li^1^, Lei Zhang^1^, Chaoqun Han^1^, Shuangning Song^1^, Qi He^1^, Ying Zhao^4^, Qingjing Zhu^4^, Hua Chen^5^, Bernd Schnabl^6^, Ling Yang*^1^, Xiaohua Hou*^1^

Author Affiliations: ^1^Division of Gastroenterology, Union Hospital, Tongji Medical College, Huazhong University of Science and Technology, 1277 Jiefang Avenue, Wuhan 430022, China

^2^ Department of Integrated Chinese and Western Medicine, Union Hospital, Tongji Medical College, Huazhong University of Science and Technology, 1277 Jiefang Avenue, Wuhan 430022, China

^3^College of Informatics, Huazhong Agricultural University, Wuhan,430070, China

^4^Liver and Infectious Diseases Department, Wuhan Jinyintan Hospital, Wuhan, China, Dongxihu District, Wuhan 430023

^5^ Tuberculosis and Respiratory Department, Wuhan Jinyintan Hospital, Wuhan, China, Dongxihu District, Wuhan 430023

^6^Department of Medicine, University of California San Diego, La Jolla, CA, USA

^#^ Huikuan Chu and Tao Bai contributed equally to this paper.

***** Ling Yang and Xiaohua Hou shared co-coresponding authors.

*** Correspondence to:**

Ling Yang, M.D., Ph.D. Division of Gastroenterology, Union Hospital, Tongji Medical College, Huazhong University of Science and Technology, 1277 Jiefang Avenue, Wuhan, 430022, China.

Email: hepayang@163.com;

phone +86-2785726678, +8613971178791

Xiaohua Hou, M.D., Ph.D. Division of Gastroenterology, Union Hospital, Tongji Medical College, Huazhong University of Science and Technology, 1277 Jiefang Avenue, Wuhan, 430022, China.

Email: houxh@hust.edu.cn

**Supplementary Table 1. Inflammatory cytokines level in liver dysfunction patients with COVID-19**

| Variables | | Normal liver function |  | Liver dysfunction | *P* values |
| --- | --- | --- | --- | --- | --- |
|  |  | median (IQR) |  | median (IQR) |  |
| IL-2 | | 2.86 (2.49~3.12) |  | 2.71 (2.34~2.82) | 0.123 |
| IL-4 |  | 2.58 (1.64~3.34) |  | 1.98 (1.74~2.12) | 0.159 |
| IL-6 | | 8.20 (4.92~10.98) |  | 8.70 (6.54~13.30) | ** |
| IL-8 |  | 10.10 (5.70~17.35) |  | 12.50 (6.45~26.65) | 0.217 |
| IL-10 | | 5.00 (5.00~5.00) |  | 5.00 (3.98~5.40) | 0.813 |
| TNF-α | | 6.30 (5.00~10.60) |  | 9.70 (7.80~16.00) | ** |

Note: ***P*<0.01. IL-2, [Interleukin 2](https://www.ncbi.nlm.nih.gov/gene/3569); IQR, interquartile range.

**Supplementary Table 2. Relationship between liver injury pattern and clinical/laboratory characteristics**

| Variables | Hepatocellular pattern  (n=48) | Cholestatic pattern  (n=170) | Mixed pattern  (n=211) | *P_for_* _all_ | *P_1_* | *P_2_* | *P_3_* |
| --- | --- | --- | --- | --- | --- | --- | --- |
|  | n (%) or median (IQR) | n (%) or median (IQR) | n (%) or median (IQR) |  |  |  |  |
| Sex |  |  |  | 0.421 | - | - | - |
| Female | 13 (27.1) | 58 (34.1) | 78 (37.0) |  |  |  |  |
| Male | 35 (72.9) | 112 (65.9) | 133 (63.0) |  |  |  |  |
| Age (years) | 58 (47~68) | 62 (50~70) | 61 (49~68) | 0.315 | - | - | - |
| Severe | 23 (47.9) | 50 (29.4) | 68 (32.2) | 0.053 | - | - | - |
| Hypertension | 21 (43.8) | 64 (37.6) | 77 (36.5) | 0.645 | - | - | - |
| Diabetes | 10 (20.8) | 28 (16.5) | 33 (15.6) | 0.682 | - | - | - |
| Coronary heart disease | 8 (16.7) | 15 (8.8) | 18 (8.5) | 0.205 | - | - | - |
| Chronic kidney disease | 1 (2.1) | 7 (4.1) | 4 (1.9) | 0.404 | - | - | - |
| Malignant tumor | 2 (4.2) | 7 (4.1) | 11 (5.2) | 0.868 | - | - | - |
| Blood oxygen saturation (%) | 94 (88~97) | 96 (92~98) | 96 (91~98) | 0.048 | 0.092 | * | 1.000 |
| White blood cell (10^9^/L) | 7.5 (4.8~10.3) | 6.4 (4.6~9.4) | 6.5 (4.9~8.4) | 0.452 | - | - | - |
| Lymphocyte (10^9^/L) | 0.8 (0.5~1.2) | 1.0 (0.7~1.4) | 1.0 (0.6~1.5) | 0.115 | - | - | - |
| Neutrophils (10^9^/L) | 5.4 (3.2~7.8) | 6.1 (3.5~9.8) | 4.7 (3.4~6.8) | 0.253 |  |  |  |
| Hemoglobin (g/dl) | 131 (120~138) | 129 (118~140) | 130 (119~140) | 0.864 |  |  |  |
| Platelets (10^9^/L) | 199 (154~313) | 189 (138~263) | 215 (161~292) | 0.025 | 0.681 | 1.000 | * |
| Albumin (g/L) | 30.8 (27.3~34.0) | 31.1 (27.7~35.6) | 30.6 (26.8~35.2) | 0.282 | - | - | - |
| Creatinine (μmol/L) | 76.9 (65.6~93.0) | 70.8 (59.1~87.9) | 71.2 (59.9~88.6) | 0.268 | - | - | - |
| Prothrombin time (s) | 13.2 (12.3~14.3) | 13.0 (11.8~14.3) | 13.2 (12.2~14.3) | 0.631 | - | - | - |
| Activated partial thromboplastin time (s) | 35.8 (31.9~39.0) | 37.6 (31.7~41.4) | 36.3 (33.5~39.4) | 0.661 | - | - | - |
| D-dimer (mg/L) | 0.93 (0.42~2.62) | 0.85 (0.43~4.94) | 0.72 (0.41~2.33) | 0.308 | - | - | - |
| International normalized ratio | 0.99 (0.94~1.06) | 1.01 (0.94~1.13) | 1.00 (0.93~1.09) | 0.389 | - | - | - |
| C-reactive protein (mg/L) | 40.8 (14.2~102.1) | 46.4 (8.9~104.9) | 39.2 (8.0~79.6) | 0.375 | - | - | - |
| Procalcitonin (ug/L) | 0.12 (0.05~0.41) | 0.09 (0.05~0.23) | 0.09 (0.05~0.23) | 0.392 | - | - | - |
| Ferritin (ug/L) | 1171.8 (601.8~2000.0) | 636.5 (351.9~1543.7) | 579.8 (284.1~1257.8) | 0.007 | 0.097 | ** | 0.508 |
| Troponin I (μg/L) | 5.7 (2.0~30.2) | 6.0 (2.2~21.6) | 5.1 (2.5~16.5) | 0.965 | - | - | - |
| Creatine Kinase (U/L) | 106 (55~282) | 89 (54~199) | 109 (52~235) | 0.499 | - | - | - |
| Cardiac injury | 12 (25.0) | 32 (18.8) | 33 (15.6) | 0.290 | - | - | - |
| Kidney injury | 5 (10.4) | 10 (5.9) | 5 (2.4) | 0.027 | 0.330 | * | 0.080 |
| IL-6 (pg/ml) | 8.47 (5.01~33.94) | 8.68 (6.97~12.70) | 8.91 (5.97~13.66) | 0.865 | - | - | - |
| Systemic inflammatory response syndrome | 19 (39.6) | 47 (27.6) | 48 (22.7) | 0.054 | - | - | - |

Note: **P*<0.05, ***P*<0.01. ULN, upper limit of normal. *P_1_*, Hepatocellular pattern vs. Cholestatic pattern; *P_2_*, Hepatocellular pattern vs. Mixed pattern; *P_3_*, Cholestatic pattern vs. Mixed pattern; IQR, interquartile range.

**Supplementary Table 3. On admission blood platelets count, albumin and coagulation levels of survival and deceased patients with COVID-19.**

| Variables | Survival |  | Deceased | P values |
| --- | --- | --- | --- | --- |
|  | n (%) or median (IQR) |  | n (%) or median (IQR) |  |
| Platelets (10^9^/L) | 213 (168~286) |  | 145 (91~200) | *** |
| PT (s) | 12.8 (11.7~13.6) |  | 14.6 (13.5~16.7) | *** |
| APTT (s) | 36.5 (33.4~39.3) |  | 36.5 (32.7~41.8) | 0.599 |
| D-dimer (mg/L) | 0.48 (0.27~1.04) |  | 8.00 (1.85~8.00) | *** |
| INR | 0.98 (0.93~1.05) |  | 1.11 (1.01~1.27) | *** |

Note: ****P*<0.001. APTT, Activated partial thromboplastin time. INR, International normalized ratio. PT, Prothrombin time.

**Supplementary Table 4. Multivariate Cox regression analyses of blood platelets count, albumin and coagulation levels association with mortality.**

| Variables | HR* | 95%CI* | P value |
| --- | --- | --- | --- |
| Platelet count | 0.995 | 0.992-0.999 | ** |
| PT | 1.030 | 1.017-1.043 | *** |
| INR | 1.464 | 1.247-1.720 | *** |
| D-Dimer | 1.039 | 1.025-1.053 | *** |

Note: *P* values were adjusted by age, sex, on admission blood oxygen saturation, kidney injury or cardiac injury. ***P*<0.01, ****P*<0.001. INR, International normalized ratio. PT, Prothrombin time.
